# Implementing David Procedure in Latin America: Closing the Gap with High-Income Countries

**DOI:** 10.21470/1678-9741-2025-0163

**Published:** 2026-06-12

**Authors:** Gustavo Prieto, Johiner Vanegas, Jesús Guerrero, Alexandra Hurtado, Maricel Licht, Lorena Montes

**Affiliations:** 1 Department of Cardiovascular Surgery, Fundación Cardiovascular de Colombia, Floridablanca, Colombia

**Keywords:** David Procedure, Aortic Valve-Sparing Operations, Reimplantation Technique.

## Abstract

**Introduction:**

David procedure has shown to be a low-risk perioperative procedure even in challenged scenarios cases, with favorable long-term outcomes and additional benefits linked to the avoidance of prosthetic valves, including freedom from anticoagulation and reintervention, reduced risk of thromboembolic complications and endocarditis. Furthermore, an aortic valve preservation program in Colombia confers probably specific advantages to our population, considering the sociodemographic factors of middle-income countries.

**Methods:**

A retrospective analysis was conducted on the clinical and perioperative results, as well as short-term follow-up data of patients who underwent David procedure at one clinical center from Colombia between November 2021 and June 2024.

**Results:**

One hundred and three patients were treated, with a mean age of 60 years, of whom 82.3% were male. In most cases, the preoperative diagnosis was aortic root dilation, with 80% presenting severe aortic insufficiency. Also 11.6% were initially diagnosed with type A acute dissection. The 30-day mortality was 0.9%. There were no cases of perioperative myocardial infarction nor dialysis requirement. Other complications were atrial fibrillation in 29.13% and acute renal failure in 9.7%. Follow-up was completed in 97.08% of cases, with survival rates at one year of 99%. Freedom from reintervention, endocarditis, and freedom from anticoagulation at one year were 100%, 100%, and 67%, respectively.

**Conclusion:**

In our study, David procedure emerged as an effective procedure, offering potential benefits that could be particularly relevant in middle-income countries. Perioperative and follow-up outcomes were comparable to those reported in large series from high-income countries.

## INTRODUCTION

**Table:** 

Abbreviations, Acronyms & Symbols	
BAV	= Bicuspid aortic valve
BMI	= Body mass index
CABG	= Coronary artery bypass grafting
CI	= Confidence interval
CPB	= Cardiopulmonary bypass
ECMO	= Extracorporeal membrane oxygenation
EuroSCORE	= European System for Cardiac Operative Risk Evaluation
FET	= Frozen elephant trunk
IQR	= Interquartile range
LVEF	= Left ventricular ejection fraction
NYHA	= New York Heart Association
PCI	= Percutaneous coronary intervention
VSRR	= Valve-sparing root replacement

Traditional management of aortic root aneurysms involves replacement with either biological or mechanical valved conduits, as described by Bentall and de Bono^[1]^. Its inherent risks in the follow-up include hemorrhage associated with anticoagulation, thromboembolic events, endocarditis, and reintervention^[2,3]^.

In 1991, Tyrone David introduced the first aortic root reimplantation surgery with valve preservation as an alternative for patients with appropriate anatomical conditions, specifically those with aortic insufficiency resulting solely from aortic root dilation^[4]^. David procedure has demonstrated favorable outcomes in the short and long terms in specialized aortic surgery centers^[5]^. Additionally, some studies reported successful outcomes even in patients with challenging surgical conditions such as type A aortic dissection and bicuspid aortic valve (BAV)^[6]^. Despite its promise, the application of David technique has been limited due to its technical complexity and steep learning curve, which compromises its reproducibility^[7]^.

In middle-income countries, challenges related to access to medical therapy, educational disparities, and limited disease awareness create a unique context where the application of this technique is likely to offer greater benefits^[8]^. Furthermore, clinical outcomes of David procedure in LATAM are unknown and hindering direct comparisons with results reported in high-income countries. A retrospective observational study was conducted to analyze outcomes following the implementation of the David procedure surgical program at a cardiac surgery center in Latin America.

## METHODS

A retrospective cohort study was conducted on adult patients over 18 years old with aortic root aneurysm who underwent the David procedure at a high-complexity institution for cardiovascular diseases in Colombia, between November 2021 and June 2024. All procedures were performed by a single experienced surgeon specializing in aortic pathologies. David procedure was indicated in patients with aortic root aneurysm or severe aortic insufficiency, particularly those with an aortic root diameter of ≥ 45 mm, meeting surgical management criteria. In cases of BAV, this threshold could be lower (between 40 and 45 mm) to enhance repair stability and achieve a 180° commissural configuration. A geometric height > 16 mm was required for tricuspid valves, and > 20 mm for bicuspid valves. Cases requiring autologous or heterologous pericardial augmentation were not included, as such repairs were considered to have poor long-term outcomes. As a general rule, the final decision was made by the treating surgeon upon intraoperative inspection of the aortic root.

Data were collected from the cardiovascular surgery service database registered in REDCap under project PID 539^[9,10]^. To ensure data integrity, a thorough review of each patient’s medical record was conducted, carefully verifying the available information. Additionally, follow-up calls were made to patients both one month and annually to confirm the accuracy of the records and complete any potential gaps.

All patients meeting the inclusion criteria were consecutively included. The clinical and sociodemographic characteristics of the study population were determined, including variables such as age, sex, history of previous cardiac surgery, hypertension, diabetes mellitus, dyslipidemia, atrial fibrillation, smoking, chronic obstructive pulmonary disease, chronic kidney disease, history of stroke, peripheral artery disease, and previous myocardial infarction. Preoperative clinical variables were also recorded, such as the diagnosis, New York Heart Association (NYHA) functional class, ejection fraction, annular diameter, and diameter of the sinuses of Valsalva, as well as the European System for Cardiac Operative Risk Evaluation (EuroSCORE) II, type of aortic valve (bicuspid or tricuspid), and history of percutaneous coronary or aortic intervention.

Intraoperative variables analyzed included technique of intervention on the valve cusps or annulus. Also, concomitant procedures such as aortic arch replacement, mitral repair, coronary artery bypass grafting (CABG), Maze procedure, left atrial appendage occlusion, and closure of atrial septal defects were included. Cardiopulmonary bypass (CPB) and aortic cross-clamping time were also recorded. Postoperative variables, both immediate and delayed complications, included stroke, renal failure, prolonged mechanical ventilation (beyond 24 hours), mediastinitis, surgical re-exploration for bleeding, atrioventricular block, and atrial fibrillation. Additionally left ventricular ejection fraction (LVEF), length of stay in the intensive care unit, and total hospital stay were recorded. Other variables, such as residual aortic insufficiency, postoperative LVEF, and in-hospital mortality, were also considered. Acute postoperative renal failure was defined according to the Kidney Disease: Improving Global Outcomes (or KDIGO) criteria as an increase in serum creatinine by ≥ 0.3 mg/dL within 48 hours or an increase to ≥ 1.5 times baseline^[11]^. The follow-up was conducted according to our institutional protocol, which includes clinical and echocardiographic evaluations at three months and annually thereafter. A residual aortic regurgitation of grade II with central jet or less on echocardiography was considered acceptable to avoid reintervention on the valve. Additionally, phone calls were made to establish the patient’s current status. Follow-up variables included mortality, follow-up duration (in months), use of anticoagulants, endocarditis, reintervention, and aortic valve regurgitation.

A descriptive analysis of the study was performed, where qualitative variables were expressed as proportions and percentages, and medians with interquartile ranges (IQR) were used for continuous variables after evaluating normality with the Shapiro-Wilk test. Survival probability was estimated using the Kaplan-Meier method.

### Ethical Statement

This study has received approval number PI-2024016 from the Scientific Technical Committee of the Fundación Cardiovascular de Colombia on March 4, 2024, and CEI-2024-07461-2 from the Ethics Committee of the Fundación Cardiovascular de Colombia on March 15, 2024. Verbally consenting was required by each patient after surgery to take part in this.

### Surgery Technique

The access route was through a median sternotomy. In all cases, CPB was established with moderate hypothermia (32°C), and myocardial protection was achieved using retrograde and antegrade “Del Nido” cardioplegia. An additional dose was administered at 60 minutes if the procedure was anticipated to exceed 90 minutes. After aortic clamping, the ascending aorta was transected, and the aneurysmal segment was resected up to the valvular plane, preserving a 4 - 5 mm remnant of the aortic wall to facilitate subsequent reimplantation of the aortic valve into the tubular graft. The coronary buttons were dissected according to the technique described by Kouchoukos et al.^[12]^.

The David type V technique was employed^[13]^, involving the interposition of two differently sized Dacron tubular grafts to reconstruct the aortic root geometry. The proximal graft, generally measuring 32 or 34 mm in diameter, was tailored with single stitches at the aortic annulus to achieve the appropriate size. To recreate the new sinotubular junction, this graft was anastomosed to a smaller distal graft, either 26 or 28 mm in diameter. The graft diameter was determined by applying traction to the commissures to ensure proper fit and function. The commissures were elevated until the leaflets coapted adequately, establishing the diameters of both the annulus and the sinotubular junction.

**Fig. 1:**
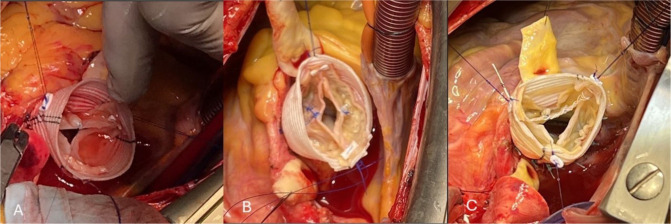
A) Free margin plication. B) Bicuspid approach. C) Gore-Tex® reinforcement.

Aortic leaflet prolapse was addressed after valve reimplantation using one or more of the following techniques: free-edge plication at the level of the nodules of Arantius (Figure 1A), subcommissural annuloplasty, raphe resection in bicuspid valves (Figure 1B), and free-edge reinforcement with Gore-Tex® sutures (Figure 1C).

Intraoperative transesophageal echocardiography was promptly performed to evaluate the final functional status. Successful management was defined as the absence of residual insufficiency or a grade I/IV insufficiency with a central jet, an effective height ≥ 9 mm, and no leaflet prolapse.

## RESULTS

A total of 103 patients were included. The median age was 60 years (IQR 52 - 58), with a predominance of males (86.27%). Most prevalent comorbidities were hypertension (51.46%), prior myocardial infarction (15.53%), atrial fibrillation (11.65%), and chronic kidney disease (5.83%). Emergent, elective, and urgent cases were 11.65%, 31.06%, and 57.28% of the included patients, respectively. Surgical indication was an ascending aortic aneurysm in 98.96% of patients, most of them presented with severe aortic insufficiency (80.58%), additionally acute type A aortic dissection was the underlying indication of surgery in 11.65% of individuals. According to the NYHA classification, most patients were in class II (47 [46.08%]), followed by class III (35 [34.31%]) (Table 1).

**Table 1:** Baseline characteristics of patients undergoing David procedure.

Variable	Category	n (103)	%
Age (years)^*^		60 (52 - 58)	
Sex	Male	90	86.27
	Female	13	13.73
Preoperative	Hypertension	53	51.46
	Smoking history	18	17.48
	Prior myocardial infarction	16	15.53
	Atrial fibrillation	12	11.65
	Dyslipidemia	9	8.74
	Diabetes mellitus	9	8.74
	Chronic kidney disease	6	5.83
	Peripheral arteriopathy	6	5.83
	Chronic obstructive pulmonary disease	3	2.91
	Previous stroke	3	2.91
	Current smoker	1	0.97
	Ascending aortic aneurysm	101	98.06
	Severe aortic insufficiency	83	80.58
	Bicuspid aortic valve	28	27.45
	Acute aortic dissection, Type A	12	11.65
	Prior history of PCI	6	5.83
	Previous cardiac surgery	2	1.94
	NYHA		
	I	6	5.88
	II	47	46.08
	III	35	34.31
	IV	14	13.73
	BMI (Kg/m^2^)^*^	20.32 (18.45 - 22.85)	
	LVEF (%)^*^	50 (40 - 58)	
	Diameter of the annulus (mm)^*^	28 (26 - 30)	
	Diameter of the sinus of Valsalva (mm)^*^	50 (48 - 60)	
	Diameter of the aortic root graft (mm)^*^	32 (32 - 32)	
	Diameter of the ascending aortic graft (mm)^*^	26 (26 - 28)	
	EuroSCORE II^*^	7.11 (4.37 - 11.05)	

* Median (percentile 25 - 75)

BMI=body mass index; EuroSCORE=European System for Cardiac Operative Risk Evaluation; LVEF=left ventricular ejection fraction; NYHA=New York Heart Association; PCI=percutaneous coronary intervention

In terms of preoperative clinical parameters, the median LVEF was 50% (IQR 40 - 58). BAV was present in 27.45% of patients. Other anatomical data included aortic annulus diameter (28 mm, IQR 26 - 30), sinuses of Valsalva diameter (50 mm, IQR 48 - 60 mm), aortic root graft diameter (32 mm, IQR 32 - 32), and ascending aorta graft diameter (26 mm, IQR 26 - 28). Additionally, 5.83% of patients had prior history of percutaneous coronary intervention, and 1.94% of previous cardiac surgeries (redo). Finally, the EuroSCORE II had a median of 7.11% (IQR 4.37 - 11.05) (Table 1).

Regarding intraoperative outcomes, 62.14% of patients required aortic cusps interventions. Most common repair techniques were: free margin plication (85.93%), raphe triangular resection of the conjoined cusp (28.13%), Gore-Tex® reinforce of free margin (7.81%), and subcommissural plasty or plication (3.13%) (Table 2). Almost half of patients (48.54%) required concomitant procedures. Aortic arch surgery was performed in 13.6%, CABG in 21.36%, mitral valve repair in 11.65%, Maze procedure and left atrial appendage closure were conducted in 8.74% and 16.5%, respectively, while atrial septal defect closure was performed in 7.77% of the cases. The median CPB time was 148 minutes (IQR 125 - 185), and the median aortic cross-clamping time was 120.5 minutes (IQR 103 - 140) (Table 3).

**Table 2:** Valve repair techniques.

Variable	Category	n (64)	%
Valve repair technique	Free margin plication	55	85.93
	Raphe triangular resection	18	28.13
	Gore-Tex® reinforce	5	7.81
	Subcommissural plasty or plication	2	3.13
	Shaving or decalcification	1	1.56

**Table 3:** Intra and postoperative characteristics of patients undergoing David procedure.

Variable	Category	n (103)	%
Concomitant procedures		50	48.54
	CABG	22	21.36
	Left atrial appendage occlusion	17	16.5
	Aortic arch replacement	14	13.59
	FET	5	4.85
	Mitral valve repair	12	11.65
	Maze procedure	9	8.74
	Atrial septal defect closure	8	7.77
	Tricuspid valve repair	1	0.97
	Cardiopulmonary bypass time (min)^*^	148 (125-185)	
	Cross-clamping time (min)^*^	120.5 (103-140)	
Postoperative	Atrial fibrillation	30	29.13
	Mechanical ventilation > 24 hours	8	7.84
	Acute renal failure	10	9.7
	Dialysis	0	0.00
	Stroke	2	1.94
	Mediastinitis	0	0.00
	Reoperation for bleeding	2	1.94
	Atrioventricular block	1	0.97
	Postoperative ECMO	1	0.97
	30-day mortality	1	0.97
	LVEF (%)^*^	52.5 (44-55)	
	Intensive care unit (days)^*^	4 (3-5)	
	Hospital stay (days)^*^	7 (5-12)	
Follow-up	Follow-up time (months)^*^	9 (4-18)	
	Aortic insufficiency		
	0	56	56
	1	36	36
	2	8	8
	Anticoagulant use	34	33.01
	Endocarditis	0	0
	Aortic valve reintervention	0	0

* Median (percentile 25 - 75)

CABG=coronary artery bypass grafting; ECMO=extracorporeal membrane oxygenation; FET=frozen elephant trunk; LVEF=left ventricular ejection fraction

Postoperative complications included acute renal failure (9.7%), none of them required dialysis support, surgical re-exploration (1.94%), stroke (1.94%), and just one case (0.97%) required a permanent pacemaker. Also, 29.13% of patients presented atrial fibrillation and 7.84% required mechanical ventilation beyond 24 hours. No perioperative myocardial infarction nor mediastinitis cases were observed. The median postoperative LVEF was 52.5% (IQR 44 - 55). The median length of stay in the intensive care unit was four days (IQR 3 - 5), the total hospital stay had a median of seven days (IQR 5 - 12), and 30-day mortality was 0.97%.

**Fig. 2:**
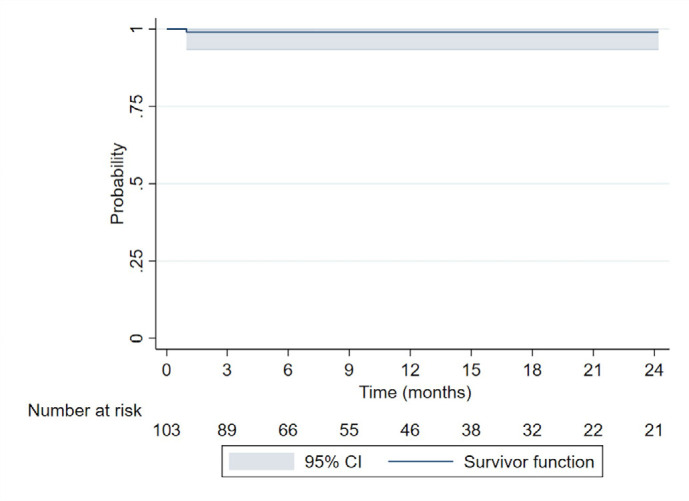
Kaplan-Meier survival curve for patients undergoing David procedure. CI=confidence interval.

Clinical and echocardiographic follow-up was completed in 97.08%, with aortic regurgitation graded as 0-I/IV in 92%, and none of them evidenced ≥ III/IV aortic insufficiency. The median follow-up time was nine months (IQR 4 - 18). According to the Kaplan-Meier survival analysis, patients who underwent the David procedure had a survival probability of 99.03% (95% confidence interval: 93.31% - 99.86%) at one month. This survival probability remained consistent at six months, one, and two years (Figure 2). Additionally, at the end of the follow-up period, 33.01% of patients were on oral anticoagulation therapy, there was 0% incidence of endocarditis, and 0% required aortic valve reintervention.

## DISCUSSION

Aortic root replacement surgery with valve preservation provides well-known benefits, avoiding the use of mechanical or biological prostheses. Additionally, it is associated with greater freedom from anticoagulation, lower risk of endocarditis, and low rates of reintervention during follow-up^[5]^. On the other hand, the reproducibility of shortand long-term results is limited due to the surgical complexity and the need of a multidisciplinary aortic team^[14]^. In our center, the valve reimplantation technique has become the cornerstone of our aortic valve preservation program. Despite the learning curve associated with this procedure, our results support its safety and efficacy, allowing us to offer this alternative in the complex surgical management of the aortic root.

Since November 2021, we have operated on 103 consecutive patients in our valve-sparing root replacement (VSRR) program, including elective surgeries, emergencies, and urgent cases. Although we encountered patients with a wide range of associated procedures, we believe this remains a selected cohort due to various factors. The experience of the surgical team, patient age, comorbidities, and risk profile are variables to consider when deciding to perform valve preservation surgery alongside other procedures or in an emergency scenario. However, it is noteworthy to achieve good results with such a high proportion of concomitant procedures (48.6%) and acute type A aortic dissections (11.5%), reflecting our philosophy of proposing the David procedure as the first strategy for the management of aortic root aneurysms, as evidenced in other series^[15]^.

We evidenced a mean age of 60 years, which is higher compared to several valve reimplantation series. Beckmann et al.^[16]^ described a mean age of 53 years in a series of 732 patients, including 16 Marfan phenotypes. T. David et al.^[17]^ reported a mean age of 46 years in their most recent series of 465 patients, of which 38 were Marfan. Although we generally use the valve preservation technique in patients with a longer life expectancy, we do not have a specific age restriction for its application, which is why, in our opinion, our cohort is somewhat older than others reported. Additionally, we have a low population of Marfan syndrome, estimated at around 4%. We cannot establish an exact diagnosis due to difficulties in accessing genetic testing for patients who do not meet clinical criteria.

This cohort exhibits a high proportion of bicuspid valve disease, comprising 27.4% of the total sample. In these cases, we performed triangular resection of the fusion raphe and, to enhance stabilization and achieve a more durable repair, we configured the commissures at 180°. In young patients, this approach was also utilized for borderline aortic diameters (40 - 45 mm)^[18,19]^. El Khoury, in a series of 68 patients with BAV undergoing VSRR with intervention on the leaflets, demonstrated a freedom from aortic regurgitation of grade II or greater and a freedom from reoperation of 95.5% at five years^[20]^. Mastrobuoni et al.^[21]^, in a meta-analysis of VSRR using the David technique with a sample of 44 studies (7,878 patients), found no significant difference at nine years of follow-up regarding freedom from reintervention between BAV and trileaflet aortic valves.

Valve reimplantation procedures themselves disrupt the geometry and configuration of the aortic root, therefore requiring intervention on the leaflets in a percentage of patients^[22]^. Additionally, our center has a non-restrictive policy regarding the management of the etiology of valve insufficiency, which is based on the classification proposed by El-Koury^[23]^. Thus, we attempt to repair valves that have leaflets with sufficient tissue, a geometric height higher than 16 mm, and being permissive regarding fenestrations that do not alter leaflet coaptation^[19]^. In our study, approximately 60% of patients required some type of intervention on the cusps.

In our cohort, the most frequently employed technique was free edge plication (85.93%), which is reported as effective and reproducible without compromising the long-term durability of the repair^[22,24]^. Other techniques were reinforcing the free edge with Gore-Tex® (7.81%) to maintain leaflet tension strength or mitigate the effects of fenestrations^[25]^, decalcification or shaving of the leaflets (1.56%) to reduce calcification and improve mobility, and partial commissural closure or subcommissural plication (3.13%), which was only utilized in combination with other techniques due to their less reliable long-term outcomes^[26]^. Finally, we do not promote leaflet expansions with pericardial patches or other materials, given their high tendency for early failure (up to 20%) within the first few years^[27]^.

In the present study, 1.94% of the patients who underwent the David procedure had a history of previous cardiac surgery. This is a low percentage, even though prior sternotomy was not considered a contraindication for performing a valve preservation procedure. Beckman et al.^[28]^, in a series of 544 elective patients, compared 30 redo patients to 514 patients with first-time sternotomy, finding that the early postoperative outcome was comparable between the redo and the first-time sternotomy groups, despite significantly more concomitant total arch replacements in the redo group. They conclude that VSRR can be performed in redo cardiac surgery without compromising the early postoperative outcome.

Our postoperative results are comparable to those described in the literature^[29]^. We did not observe any cases of mediastinitis and reported a low incidence of stroke, atrioventricular block, and reoperation due to bleeding, at 1.94%, 0.97%, and 1.94%, respectively. On the other hand, our postoperative rate of atrial fibrillation is almost 30%. T. David, in his series of 465 patients with an average follow-up of 10 years, reported postoperative complications including stroke at 0.6%, the need for permanent pacemaker implantation at 1.9%, reoperation due to bleeding at 7.3%, and De Novo atrial fibrillation at 23.4%^[17]^. Lastly, during our study period, there was only one death within the first 30 days secondary to a hemorrhagic stroke in a patient with aortic root aneurysm without valve insufficiency and severe ischemic ventricular dysfunction. The patient had a complicated postoperative course with episodes of recurrent atrial fibrillation and the need for anticoagulant therapy. Those results highlight that with the appropriate learning curve and training, it is possible to reproduce good results from the valve reimplantation technique and promote the establishment of centers of excellence for aortic root management in middle-income countries like ours.

The average follow-up duration in our cohort was nine months, with no observed mortality, reoperations, or cases of endocarditis. Additionally, 92% of patients demonstrated aortic regurgitation grades of 0-I/IV, with no cases exceeding grade II/IV among those who completed echocardiographic follow-up. Our follow-up protocol includes annual clinical and echocardiographic evaluations. By the cutoff date of this study, 20% of patients had surpassed two years post-intervention, demonstrating stability in valve function.

No aortic valve re-interventions were reported. However, one patient required aortic arch replacement with a frozen elephant trunk due to symptomatic Non A-Non B aortic dissection occurring three months after the David procedure. While these results are encouraging, such interventions require long-term monitoring to evaluate the durability and stability of valve repair.

Freedom from anticoagulation was observed in 67% of patients in one year. Although aortic valve preservation itself does not require anticoagulation, these figures are influenced by the occurrence of atrial fibrillation during follow-up. However, the use of direct oral anticoagulants in this context is feasible, facilitating improved outpatient management and monitoring, an option that would not be available with a mechanical prosthesis. We are not surprised by the increase in the incidence of atrial fibrillation during follow-up, as preoperative data indicated a prevalence of 11%. However, most of our patients come from rural areas and did not have proper medical management prior to surgery, and the possibility of undiagnosed paroxysmal atrial fibrillation before the procedure cannot be ruled out.

In middle-income countries like Colombia, we believe that valve preservation procedures could offer greater benefits for our patients. The demographic and socioeconomic conditions of our region limit access to healthcare systems and complicate adherence to anticoagulant treatment, with adherence to anticoagulant therapy using coumarins estimated at only 40% in middleand low-income countries^[8]^. In this context, the use of techniques aimed at avoiding anticoagulation and the non-use of valve prostheses is particularly appealing in our setting.

### Limitations

Several limitations are inherent to retrospective analyses. This study is restricted by its single-surgeon design. Selection bias may also be present, as the decision to perform David procedure was based on the surgeon's clinical judgment. Furthermore, the small sample size, particularly in comparison to larger studies, underscores the need for further research, particularly in middle-income countries.

## CONCLUSION

In conclusion, our series of valve reimplantation surgery shows excellent perioperative and short-term results, comparable to large series described in the literature. It is necessary to continue prospective follow-up of our patients in the medium and long terms. We are convinced that the potential benefits of these techniques are even greater in the context of middle-income countries.

## Data Availability

The authors declare that the data supporting the findings of this study are available within the article.

## Artificial Intelligence Usage

The authors declare use of ChatGPT (OpenAI, San Francisco, CA, USA) to assist with language editing and manuscript refinement The content produced by the artificial intelligence tool was revised and edited by the authors as necessary, and they take full responsibility for the content to be published.

## References

[1] Bentall H, De Bono A. (1968). A technique for complete replacement of the ascending
aorta. Thorax.

[2] Chan V, Malas T, Lapierre H (2011). Reoperation of left heart valve bioprostheses according to age at
implantation. Circulation.

[3] Gocoł R, Bis J, Malinowski M (2022). Outcome comparison of different approaches to aortic root
aneurysm. Kardiol Pol.

[4] David TE, Feindel CM. (1992). An aortic valve-sparing operation for patients with aortic
incompetence and aneurysm of the ascending aorta. J Thorac Cardiovasc Surg.

[5] David TE. (2024). Aortic valvesSparing operations. Ann Thorac Surg.

[6] Irimie V, Atieh A, Kucinoski G (2020). Long-term outcomes after valve-sparing anatomical aortic root
reconstruction in acute dissection involving the root. J Thorac Cardiovasc Surg.

[7] Beckmann E, Martens A, Krueger H (2020). Aortic valve-sparing root replacement (David): learning curve and
impact on outcome. Interact Cardiovasc Thorac Surg.

[8] Ramakumar V, Benz AP, Karthikeyan G. (2021). Long-term oral anticoagulation for atrial fibrillation in low and
middle income countries. Indian Heart J.

[9] Harris PA, Taylor R, Minor BL (2019). The REDCap consortium: Building an international community of
software platform partners. J Biomed Inform.

[10] Harris PA, Taylor R, Thielke R (2009). Research electronic data capture (REDCap)--a metadata-driven
methodology and workflow process for providing translational research
informatics support. J Biomed Inform.

[11] Kellum JA, Romagnani P, Ashuntantang G, Ronco C, Zarbock A, Anders HJ. (2021). Acute kidney injury. Nat Rev Dis Primers.

[12] Kouchoukos NT, Marshall WG (1986). Wedige-Stecher TA. Eleven-year experience with composite graft
replacement of the ascending aorta and aortic valve. J Thorac Cardiovasc Surg.

[13] Cameron DE, Alejo DE, Patel ND (2009). Aortic root replacement in 372 Marfan patients: evolution of
operative repair over 30 years. Ann Thorac Surg.

[14] Isselbacher EM, Preventza O, Hamilton Black J (2022). 2022 ACC/AHA Guideline for the Diagnosis and Management of Aortic
Disease: A Report of the American Heart Association/American College of
Cardiology Joint Committee on Clinical Practice Guidelines. Circulation.

[15] Settepani F, Bergonzini M, Barbone A (2009). Reimplantation valve-sparing aortic root replacement with the
Valsalva graft: what have we learnt after 100 cases?. Interact Cardiovasc Thorac Surg.

[16] Beckmann E, Martens A, Krüger H (2021). Aortic valve-sparing root replacement with Tirone E. David’s
reimplantation technique: single-centre 25-year experience. Eur J Cardio-Thorac Surg Off J Eur Assoc Cardio-Thorac Surg.

[17] David TE, David CM, Ouzounian M (2021). A progress report on reimplantation of the aortic
valve. J Thorac Cardiovasc Surg.

[18] Ehrlich T, De Kerchove L, Vojacek J (2020). State-of-the art bicuspid aortic valve repair in
2020. Prog Cardiovasc Dis.

[19] Schäfers HJ. (2019). The 10 commandments for aortic valve repair. Innovations (Phila).

[20] El Khoury G, Vanoverschelde JL, Glineur D (2006). Repair of bicuspid aortic valves in patients with aortic
regurgitation. Circulation.

[21] Mastrobuoni S, Govers PJ, Veen KM (2023). Valve-sparing aortic root replacement using the reimplantation
(David) technique: a systematic review and meta-analysis on survival and
clinical outcome. Ann Cardiothorac Surg.

[22] David TE, David CM, Feindel CM (2017). Reimplantation of the aortic valve at 20 years. J Thorac Cardiovasc Surg.

[23] El Khoury G, Glineur D, Rubay J (2005). Functional classification of aortic root/valve abnormalities and
their correlation with etiologies and surgical procedures. Curr Opin Cardiol.

[24] Settepani F, Cappai A, Raffa GM (2015). Cusp repair during aortic valve-sparing operation: technical
aspects and impact on results. J Cardiovasc Med Hagerstown Med.

[25] David TE, Armstrong S. (2010). Aortic cusp repair with Gore-Tex sutures during aortic
valve-sparing operations. J Thorac Cardiovasc Surg.

[26] Fattouch K, Castrovinci S, Murana G (2014). Functional annulus remodelling using a prosthetic ring in
tricuspid aortic valve repair: mid-term results. Interact Cardiovasc Thorac Surg.

[27] Karliova I, Schneider U, Ehrlich T (2020). Results of Pericardial Patches in Tricuspid and Bicuspid Aortic
Cusp Repair. Ann Thorac Surg.

[28] Beckmann E, Kaufeld T, Martens A (2022). Is aortic valve-sparing root reimplantation (David-I) justified
in cardiac redo surgery?. Interact Cardiovasc Thorac Surg.

[29] Kallenbach K, Hagl C, Walles T (2002). Results of valve-sparing aortic root reconstruction in 158
consecutive patients. Ann Thorac Surg.

